# Effect of Different Extraction Methods and Geographical Origins on the Total Phenolic Yield, Composition, and Antimicrobial Activity of Sugarcane Bagasse Extracts

**DOI:** 10.3389/fnut.2022.834557

**Published:** 2022-02-24

**Authors:** Victor Velázquez-Martínez, Delia Valles-Rosales, Laura Rodríguez-Uribe, Juan Rodrigo Laguna-Camacho, Hector Daniel López-Calderón, Efren Delgado

**Affiliations:** ^1^Industrial Engineering, New Mexico State University, Las Cruces, NM, United States; ^2^Department of Family and Consumer Sciences, New Mexico State University, Las Cruces, NM, United States; ^3^Facultad de Ingeniería Mecánica Eléctrica, Universidad Veracruzana, Veracruz, Mexico; ^4^Plant and Environmental Sciences, New Mexico State University, Las Cruces, NM, United States; ^5^Instituto de Biotecnología, Universidad Autónoma de Nuevo Leon, Nuevo Leon, Mexico

**Keywords:** bioactive compounds, antimicrobial activity, agro-industrial byproducts, anticarcinogenic properties, *Enterobacter aerogenes*, *Streptococcus aureus*, *Enterobacter cloacae*

## Abstract

Several parameters, including particle size, solvent, temperature, and extraction method, affect phenolic compounds' extraction yield from a plant matrix. Considering the wide availability of sugarcane bagasse (SCB), this study analyzed the effect of different extraction methods and geographical origins on the yield, quality, and antimicrobial activity of phenolic compounds from SCB extracts. Samples from three geographical locations (Veracruz, Mexico; Santa Rosa, Texas, USA; and St. Mary, Louisiana, USA) were analyzed. Extraction was performed using an orbital shaker or ultrasonic bath at various times at a fixed temperature of 50°C, with 90% ethanol or methanol. The highest yield (5.91 mg GAE) was obtained using an orbital shaker for 24 h with 90% methanol as the solvent. HPLC-MS identified desferrioxamine b, baicalein, madecassic acid, and podototarin at different concentrations in all three SCB samples. The antimicrobial activity of these compounds was tested against *Escherichia coli K12, Bacillus cereus, Enterobacter aerogenes, Streptococcus aureus*, and *Enterobacter cloacae*. The antimicrobial activity was also tested against modifications of the *Saccharomyces cerevisiae*: the MutL Homolog 1 (MLH1), Slow Growth Suppressor (*SGS1*), O-6-MethylGuanine-DNA methyltransferase (*MGT1*), and RADiation sensitive (*RAD14*), carrying mutations related to different cancer types. In addition, the results were compared with the effect of ampicillin and kanamycin. The SCB extracts showed up to 90% growth inhibition against *B. cereus* at 200–800 μg/mL and 50% growth inhibition against *S. aureus* at 800 μg/mL. The inhibitory effect against modified yeast *SGS1*, RAD*14*, and *MLH1* was 50–80% at 800 μg/mL. The percentage of inhibition and the phenolic compound contents differed depending on the origin of the SCB sample. These findings are promising for using this industrial byproduct to obtain compounds for nutraceutical, food additive, or medical uses.

## Introduction

During harvest season, every sugar mill worldwide processes large quantities of sugarcane, generating vast amounts of bagasse as a byproduct. For example, in the United States (the 10th largest producer worldwide), Louisiana milled 15 million tons of sugarcane (1) in 2018, producing around 4 million tons of SCB. In Mexico, the state of Veracruz milled over 20 million tons of sugarcane in 2015, producing over 5 million tons of SCB (2). This byproduct can be used as a potential source of phenolic compounds (PhCs) and has beneficial antioxidant properties (3).

Several parameters, such as particle size, solvent, temperature, and extraction methods, ensure the best extraction yield of PhCs from a plant matrix. As the first step in extracting polyphenols from plants, sample grinding is crucial for obtaining the highest yield of the targeted compounds. In addition, a smaller particle size offers a greater surface area, allowing better interaction of the chosen solvent with the samples; however, this may not always be true because tiny particles can also lead to aggregation, affecting the overall extraction.

The combination of different parameters (particle size, temperature, method, and solvent) can result in different extracted PhCs. The most common PhCs extraction methods include Soxhlet extraction, maceration, supercritical CO_2_, and sonication (4). The type of solvent used is also essential because PhCs have different chemical properties and polarities that can affect their solubility. Polar solvents are preferred solvents to extract PhCs from plant matrices, among which ethanol is a suitable solvent and is safe for human consumption. On the other hand, methanol is better to extract polyphenols with low molecular weights, and acetone is suitable for flavanols (5).

The temperature must also be controlled to preserve the PhCs while drying the plant matrix and extraction. Increasing temperature has been correlated with the loss of compounds and their antioxidant activity (6). As every plant is unique, intensive research has been undertaken to determine the best parameter combination for PhCs extraction. For example, for extraction from *Guiera senegalensis*, 50% ethanol and 70% ethanol using an ultrasonic water bath for 2 h were reported as the best solvents and conditions (7). However, for Pinus densiflora Bark, 20% ethanol, 40% ethanol, or 20% acetonitrile at 60°C for 9 h in an isomantle resulted in the highest PhCs extraction (8).

PhCs has been extracted from sugarcane juice using methanol: ammonia (99.5:0.5 v/v), followed by concentration at 40°C using roto-evaporation (9). PhCs from sugarcane has also been extracted using 50% ethanol followed by fractionation with ethyl acetate (10) or 60% ethanol using an ultrasonic bath at 60°C (11).

The PhCs in sugarcane varies because of the extraction and phytochemical profiles (sugarcane wax, juice, and leaves) and its products. For example, sugarcane wax contains long-chain fatty alcohols and fatty acids, various phytosterols, steroids, and high levels of terpenoids (12). The sugarcane juice color was previously assumed to be attributed to plant pigments. However, it was recently discovered to be derived from different components classified into four major classes: plant pigments, polyphenolic compounds, caramels, and degradation products of sugars condensed with amino derivatives (12). Luteolin, tricin, apigenin, caffeic acid, hydroxycinnamic acids, and sinapic acid have been reported to be present in sugarcane juice (9). HPLC micro fractionation of the methanolic extract of sugarcane leaves successfully revealed various flavones –O– and –C– glycosides (12). Sugarcane tops, including leaves and immature cane, have also been reported to contain PhCs such as apigenin, caffeic acid, hydroxycinnamic acid, albanin A, quercetin, australone A, and moracin M (13). Mill syrups, brown sugar, molasses, and non-centrifugal sugar are byproducts of sugar processing from sugarcane. In addition to the PhCs mentioned above, intensive research has identified three compounds, tricin7-(2′-rhamnosyl)-α-galacturonide, orientin-7, 3′-dimethyl ether, and iso-orientin-7,3′-O-dimethyl ether (12), from these byproducts. The compounds genistin, p-coumaric acid, genistein, and quercetin have been identified by ethanolic extraction of SCB (11).

The PhCs presence and its concentration are also affected by several factors such as its geographical location, rainfall, temperature, microorganisms, or herbivore exposure. For example, plants produce flavonoids to protect them from UV rays, while anthocyanins production is related to temperature. Therefore, the UV index in different locations and the average temperature can cause differences in the concentration of the metabolites mentioned above (10). In another study, green tea showed more total catechins at higher elevations and high content of free amino acids at lower temperatures (14). The secondary metabolites are also related to plants flowering, such as flavonoids, PhCs, and alkaloids, to name a few. For example, in comparing sugarcane flowering, higher temperature, and low precipitation negatively affected the floral stimulus of sugarcane (15).

Microbial bioassays can be used to estimate the activity of a substance to stimulate or inhibit the growth of a microbial test organism. A microbial bioassay requires practical and fully characterized microbial strains. Culturable and non-culturable techniques are used to identify and characterize microbial strains. The agar diffusion method, widely used in antibiotic assays, relates the size of the zone of inhibition to the antibiotic assay dose (16). The technique performed in 96-well-microplates required fewer extracts and produced reproducible results to determine the minimal inhibitory concentrations of the possible bactericidal or bacteriostatic effects (17). A previous study successfully tested SCB extracts against bacterial strains using 96-well-microplates. The pigment MTT was used as a growth indicator to avoid the interfering of microorganisms cells aggregation, the sugarcane extracts color, and compounds precipitation (3, 17).

Few studies have analyzed the microencapsulating potential of SCB phenolic compounds that can be used as additives in functional foods. This research's novelty lies in utilizing SCB waste as a new source of phenolic compounds and their comparison between three regions and two different countries. Furthermore, this research aims to determine the effect of different extraction methods and geographical origins on the TPC yield, composition, and characteristics of SCB extracts and their antimicrobial effects against different bacterial strains and modified yeast.

## Materials and Methods

### Sugarcane Bagasse Sample Preparation

The SCB was obtained from three locations: (a) Mahuixtlan, Ver, Mexico, (b) Santa Rosa, TX, US, and (c) St. Mary, LA, US. All three samples were oven-dried (Blue M oven, Lindberg/MPH, Riverside, MI, USA) at 55°C for 24 h. After drying, the samples were ground using a Wiley mill Model 4 (Thomas Scientific, Swedesboro, NJ, USA) and passed through a 500 μm sieve. All samples were vacuum sealed and stored at −20°C until further use.

### Chemicals

Gallic acid was purchased from ACROS Organics (ACROS Organics, Geel, Belgium). Sodium carbonate anhydrous ACS reagent was purchased from Sigma-Aldrich (Sigma-Aldrich, St. Louis, MO, USA). Folin-Ciocalteu was purchased from MP Biomedicals (MP Biomedicals, LLC, Irvine, CA, USA). Ethanol and methanol were purchased from Alfa Aesar (Alfa Aesar, Tewksbury, MA, USA). HPLC grade methanol and MTT tetrazolium were purchased from MilliporeSigma (MilliporeSigma, Burlington, MA, USA). Other chemicals used were of analytical grade and were obtained from Alfa-Aesar.

### Nutrient Composition of Sugarcane Bagasse Samples

The nutrient composition of SCB samples was assessed using the following methods. Ash content by AOAC 942.05, the minerals were determined by digestion of samples in the microwave and inductively coupled plasma spectrometer (AOAS 2011.14). The lignin content by filter bag technique, method 9, and AOAZ 973.18.

### Analysis of Extraction Methods of Bioactive Compounds

The combination of parameters (temperature, method, and solvent) can lead to different results on the yield of extracted PhCs. The most common extraction methods of PhCs from plants are soxhlet, maceration, supercritical CO_2_, and sonication (4). The extraction methods of PhCs selected were sonication and orbital shaker because they do not need constant supervision, are easy to handle, and are affordable. The choice of solvents was methanol and ethanol because of their low cost, low risk, and proven in PhCs extraction; the ratio solvent-water ranged from 40 to 80% in several studies (4, 11, 13, 18–20). Therefore 90% was selected to give a new insight. Finally, the temperature was fixed to 50°C as a middle point among the temperatures used in other studies to preserve the extracted PhC and its antioxidant activity (9, 11, 21).

For the extraction of phenolic compounds, a Central Composite Experimental Design, with a quadratic model was conducted (Design Expert 11, STAT-EASE INC, Minneapolis, MN, USA). Two different solvents (90% ethanol or 90% methanol) were used. Three extraction methods were used at a fixed temperature of 50°C: an ultrasonic bath for 30 min (CPXH series Heated Utrasonic Cleaning bath, ThermoFisher, Waltham, MA, USA), orbital shaker for 24 h at 120 rpm, and orbital shaker for 48 h at 120 rpm (MaxQ HP Incubated Tabletop Orbital Shaker, ThermoFisher Scientific). The experimental design table is given as Supplementary Material. One gram of sample mixed with 20 mL of either ethanol or methanol was used for every treatment condition. After extraction, all samples were centrifuged for 10 min at 3,000 × g. The supernatant was filtered through a Whatman filter paper grade 1 and stored at −4°C until further use.

### Lyophilization Sugarcane Bagasse Extracted Phenolic Compounds

The PhCs were extracted using the best treatment combination from the extraction analysis from SCB from all three locations and concentrated by rotoevaporation (Heidolph Laborota, 4000 efficient, Schwabach, Germany). After collecting the concentrated extracts, all samples were frozen with liquid nitrogen and dried by lyophilization for 48 h (FreeZone Labconco, Kansas City, MO, USA). The lyophilized powder samples were then stored at −4°C until further use.

### Total Phenolic Content

The TPC was used as a response variable to test each combination of PhCs extraction methods. The TPC was measured following the Folin-Ciocalteu reagent method used in a previous study (3). Briefly, 100 μL of extract from each treatment combination was diluted by adding 900 μL of distilled water (DI water). After, 0.5 mL of Folin-Ciocalteu reagent (previously diluted to 50% with DI water) were added to each dilution. Next, all mixtures were incubated in the dark at room temperature for 5 min. After incubation, 1.5 mL of 20% sodium carbonate and 7 mL of DI water were added to each tube and vortexed. All tubes were then incubated for 10 min at 75°C in a water bath (StableTemp, Cole-Parmer, Vernon Hills, IL, USA). The absorbance from each tube was then read using a spectrophotometer Genesys 10S UV-Vis (ThermoFisher Scientific, Waltham, MA, USA) at 760 nm against a corresponding blank reagent (either ethanol or methanol). A series of dilutions (using either 90% ethanol or 90% methanol) of gallic acid were used to obtain the standard calibration curve. The TPC values were expressed as gallic acid equivalents per gram of dry weight (GAE).

### High-Performance Liquid Chromatography

All sample suspensions were prepared using 2 mg of lyophilized powder suspended in 1 mL of HPLC grade methanol. The analysis was conducted using a mass spectrometer (QTOF Ultima, Waters, Manchester, UK) and a Waters Acquity UPLC system (Waters, Manchester, UK). The mass spectra from all samples were collected in the negative electrospray ionization mode (ESI-) using eluents A (water) and B (acetonitrile) containing 0.1% formic acid. Three different runs from each sample were analyzed and aligned in MS-dial software to identify the bioactive compounds with a tolerance of 0.1 Da for Ms1/Ms2 and a deconvolution parameter with a sigma value of 0.5 (22).

### PLS-Discriminant Analysis

The alignment results were retrieved from Ms-dial, and a database was created. Finally, the data were filtered, normalized, and subjected to PLS-DA were conducted using MetaboAnalyst (18) web interface software for metabolomics data analysis to test differences between SCB samples.

### Antimicrobial Activity of Sugarcane Bagasse Extracts

The antimicrobial activity of SCB extracts was measured using a colorimetric method (19). Briefly, 50 mg of lyophilized phenolic powder from each sample was suspended in 5 mL of DMSO: DI water mixed in a ratio of 1:1. The sample mixture was filtered through 0.25 μm sterile syringe filters and diluted to final concentrations of 0.8–800 ppm. In a microplate, 20 μL of each sample concentration was mixed with 220 μL of the corresponding broth medium to grow each microorganism and 10 μL of a particular microorganism in solution, previously measured to be between 0.1 and 0.2 absorbances at 630 nm, was added. After incubation at 30°C for 16 h, 25 μL of MTT was added to each well, and the microplate was incubated again for 1 h. The percentage of inhibition was determined by spectrophotometry in a microplate reader at 630 nm. The change in color of MTT from yellow to purple was observed, and the growth inhibition was calculated as:


(1) $$\begin{array}{l} \text{Percentage of inhibition}=100\%-\frac{A_{s}\;*\;100}{A_{t1}+\;A_{t2}} \end{array}$$

Where *A*_*s*_ is the absorbance of the sample, *A*_*t*1_ is the absorbance of the sample without microorganisms, and *A*_*t*2_ is the absorbance of the microorganism without treatment.

The effect of SCB extracts was compared with the effects of two antibiotics, Kanamycin (KAN) and ampicillin (AMP), which were prepared the same way as the samples. The bacterial strains used for this experiment were *Escherichia coli K12, Bacillus cereus, Enterobacter aerogenes, Streptococcusaureus*, and *Enterobacter cloacae*. The antimicrobial activity was also tested against modifications of the *Saccharomyces cerevisiae*: the MutL Homolog 1 (*MLH1*), Slow Growth Suppressor (*SGS1)*, O-6-MethylGuanine-DNA methyltransferase (*MGT1)*, and RADiation sensitive (*RAD14)*, carrying mutations related to different cancer types. The effect of samples and antibiotics against each microorganism was tested using the microplate technique in triplicates.

### UV Index, Temperature, and Precipitation

The UV index, temperature and precipitation values from the US locations were obtained from the United States UV Index Report (23) and the U.S. climate data (24). All data from Mexico were obtained from the Mexican government site “National Water Comission CONAGUA” (25).

### Statistical Methods

A two-way analysis of variance with blocking was conducted to test the interaction between the solvent and extraction method to extract the phenolic compounds from SCB. The statistical software used was Rstudio v.1.4.1103 (RStudio, Boston, MA, USA).

## Results and Discussion

### Analysis of Extraction Methods for Bioactive Compounds

**Figure 1:**
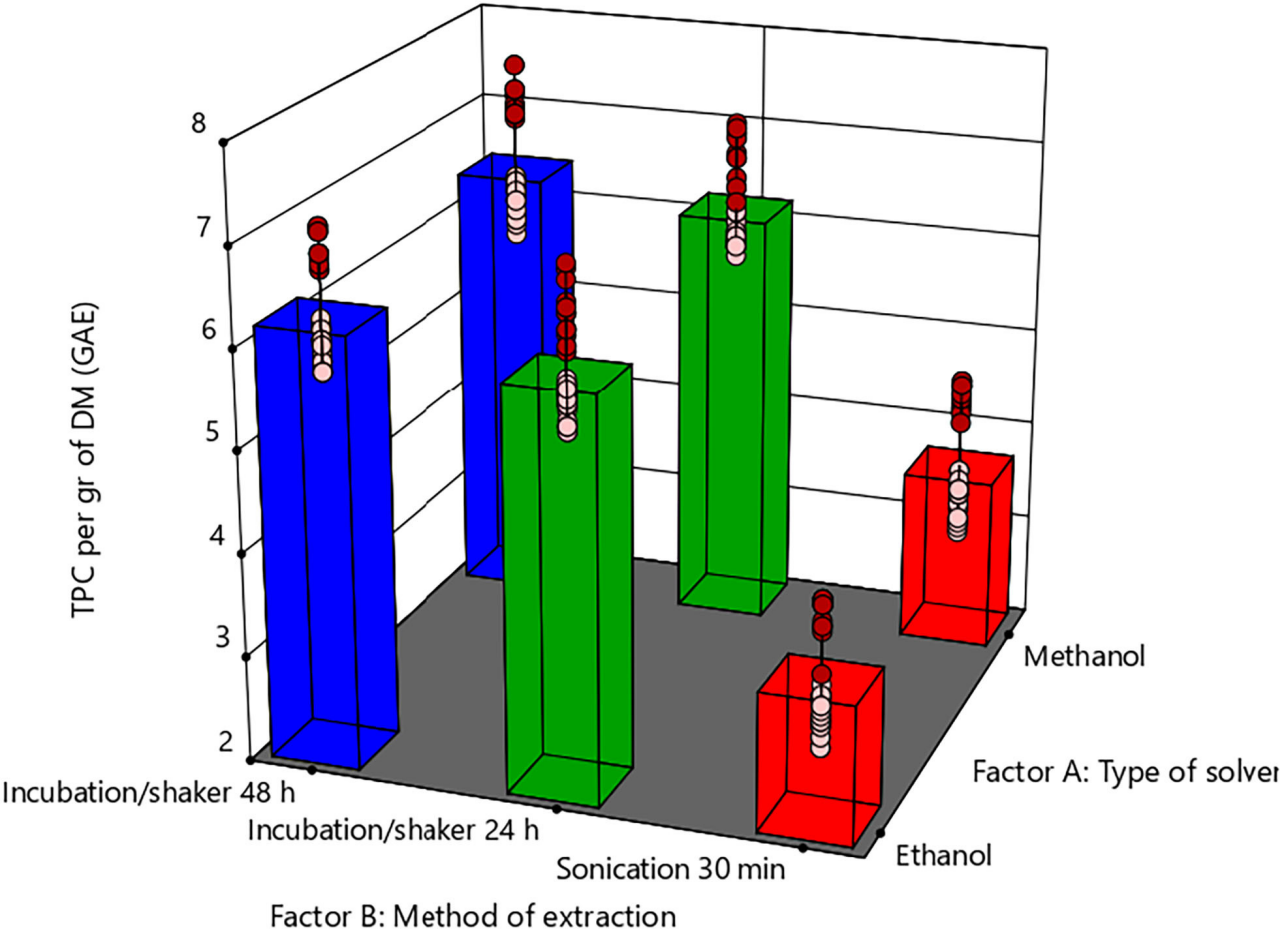
Model graph and two-way ANOVA with blocking results between factor A (type of solvent−90% ethanol or methanol) and factor B (extraction method). Pairwise comparison between best methods for extraction of phenolic compounds using methanol 90%.

Figure 1 shows the interaction effect of solvents (Factor A: 90 % ethanol or methanol) and extraction method (Factor B:1 = Sonication 30 min, 2 = Orbital shaker 24 h, 3 = Orbital shaker 24 h) on the extracted TPC. The interaction between the solvent type and extraction method was significant (*P* = 0.0148), and each of the main effects, solvent type, and extraction method was found to affect the TPC (*P* < 0.05). According to the TPC values in figure 1, methanol as a solvent extracted more PhCs than ethanol, depending on the extraction method. Sonication for 30 min offered 30 and 50% less TPC than an Orbital shaker for 24 and 48 h, respectively (Figure 1). There was no significant difference (*P* > 0.05) between the 24 h and 48 h orbital shaker treatments because the volume of the solvent used for extraction was almost saturated at the end of 48 h. Therefore, an average TPC yield of 5.91 mg GAE/g was obtained from SCB by the treatment combination of methanol and orbital shaker used for 24 h.

The TPC from SCB obtained in this study was lower than the average of 7.83 mg GAE/g reported in another study on SCB (11). However, these TPC differences could be related to the number of times the sample was exposed to the solvent. The TPC yield obtained from this study was higher than 1.78 mg/GAE and 4.26 mg/GAE obtained from SCB samples subjected to pyrolysis (26). Further, the TPC extracted from SCB was higher than the 3.91 mg GAE/g obtained from B-type molasses (27) and the 3.751 mg GAE/g from molasses retrieved from sugar mills in Pakistan (28). Compared with other industrial byproducts, this study's SCB TPC was lower than the 0.38 mg/GAE extracted from *Citrus limetta* bagasse (29). It was also lower than the TPC extracted from six different white and red wine grape wastes, which showed TPC in a range of 32–59 mg GAE/g (30). However, the total phenolic content from SCB is significant due to the raw material's easy availability.

### PLS-Discriminant Analysis

**Figure 2:**
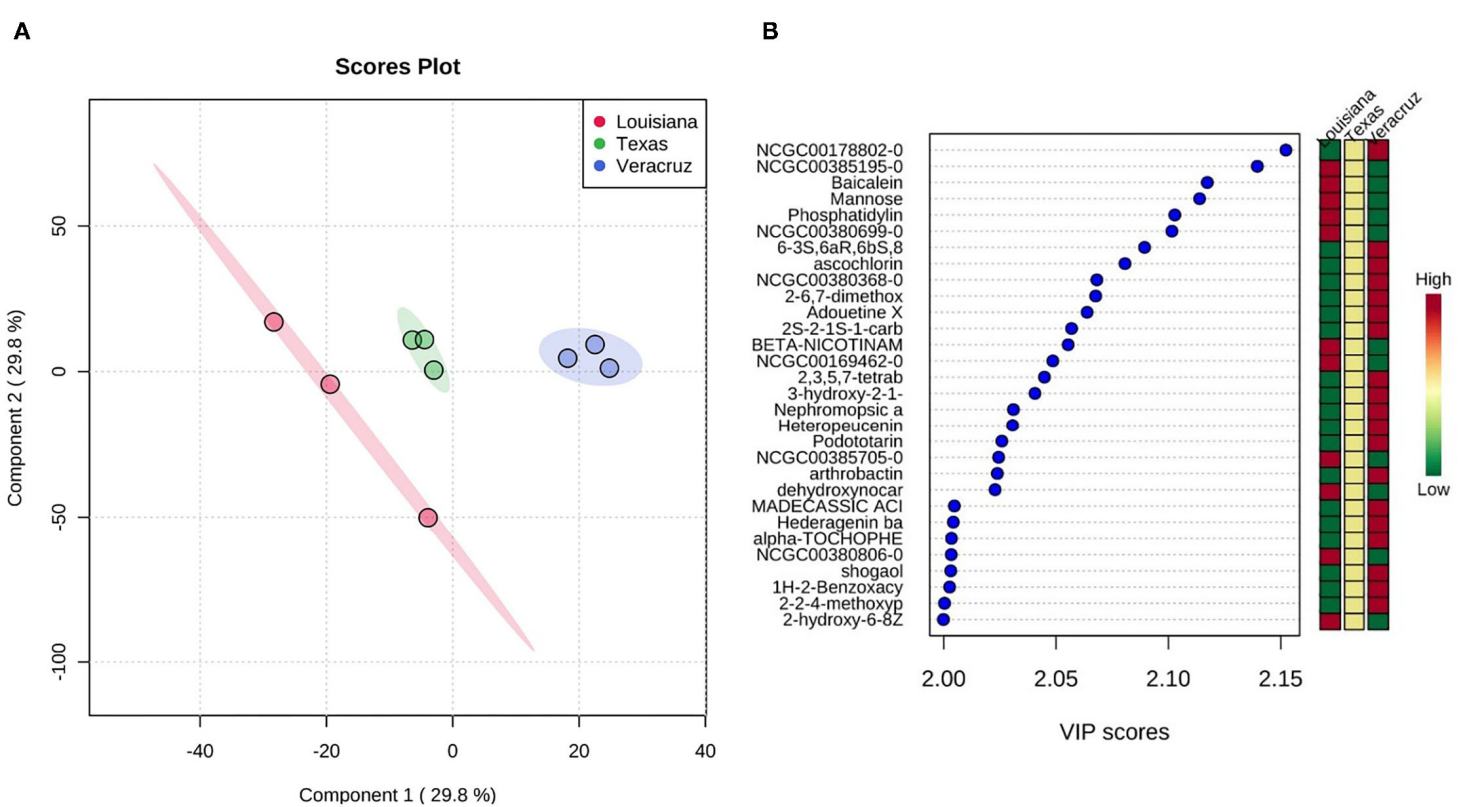
**(A)** Scores plot between the selected principal components. The explained variances are shown in brackets. **(B)** Important features identified by PLS-DA. The colored boxes on the right indicate the relative concentrations of the corresponding metabolite in each group under study.

Figure 2 shows the 2-D scores plot between selected components and the important features identified by PLS-DA. The TX sample generally showed an intermediate abundance of each compound compared with the LA and VER samples, which varied between higher or lower abundance. This variation was expected as the samples are derived from crops planted on different soils. For example, the compound NCGC00178802-0 (desferrioxamine b) was more abundant in the VER samples than LA and TX samples. This compound is also known for removing excess iron from the body as a chelating agent. One study used steep corn liquor's byproduct to produce desferrioxamine (31). Baicalein, a compound found in all three samples, is significant because it has powerful antioxidant properties that can induce programmed cell death in breast and carcinogenic ovarian cells (32). Podototarin is a bisditerpenoid that can reduce rapid growth and proliferation (33). The VER sample had a higher content of Podototarin than that in the LA and TX samples.

### Effect of Different Factors on the Presence of Secondary Metabolites in Sugarcane Bagasse Samples

The values shown in Table 1 are the results from the nutrient composition analysis carried out in a previous study.

**Table 1:** Multivariate analysis results from the nutrient composition of sugarcane bagasse samples from three different locations.

	**VER**	**LA**	**TX**	**MANOVA**
% Lignin	13.8 ± 0.21	11.10 ± 0.75	9.20 ± 0.36	^*^
% Ash	6.4 ± 0.10	7.9 ± 0.06	9.5 ± 0.10	^*^
% Calcium	0.16 ± 0.01	0.11 ± 0.01	0.49 ± 0.02	^*^
ESC (simple sugars)	0.53 ± 0.06	4.37 ± 0.31	0.50 ± 0.17	^*^
% Phosphorus	0.05 ± 0.00	0.03 ± 0.01	0.04 ± 0.00	^*^
% Magnesium	0.08 ± 0.00	0.06 ± 0.00	0.11 ± 0.00	^*^
% Potassium	0.24 ± 0.01	0.18 ± 0.01	0.28 ± 0.01	^*^
% Sodium	0.01 ± 0.00	0.01 ± 0.00	0.08 ± 0.00	^*^
ppm Iron	2103.3 ± 131	1,690.0 ± 30	2,080.0 ± 52.9	^*^
ppm Zinc	15.0 ± 0.00	14.3 ± 0.58	33.3 ± 0.58	^*^
ppm Cooper	54.7 ± 17.93	3.00 ± 0.00	5.33 ± 0.58	^*^
ppm Manganese	93.3 ± 0.58	79.3 ± 0.58	52.33 ± 1.53	^*^
ppm Molybdenum	1.10 ± 0.20	1.03 ± 0.15	0.93 ± 0.06	NS
% Sulfur	0.06 ± 0.00	0.04 ± 0.00	0.06 ± 0.00	^*^
% Chloride ion	0.07 ± 0.01	0.05 ± 0.01	0.06 ± 0.01	NS

*VER, Mahuixtlan, Ver; MX, LA, St. Mary, LA, US; TX, Santa Rosa, TX, US. ESC, ethanol soluble carbohydrates. NS, not significant different within the same line*.

* *indicates statistical differences within the same line*.

#### Temperature

**Figure 3:**
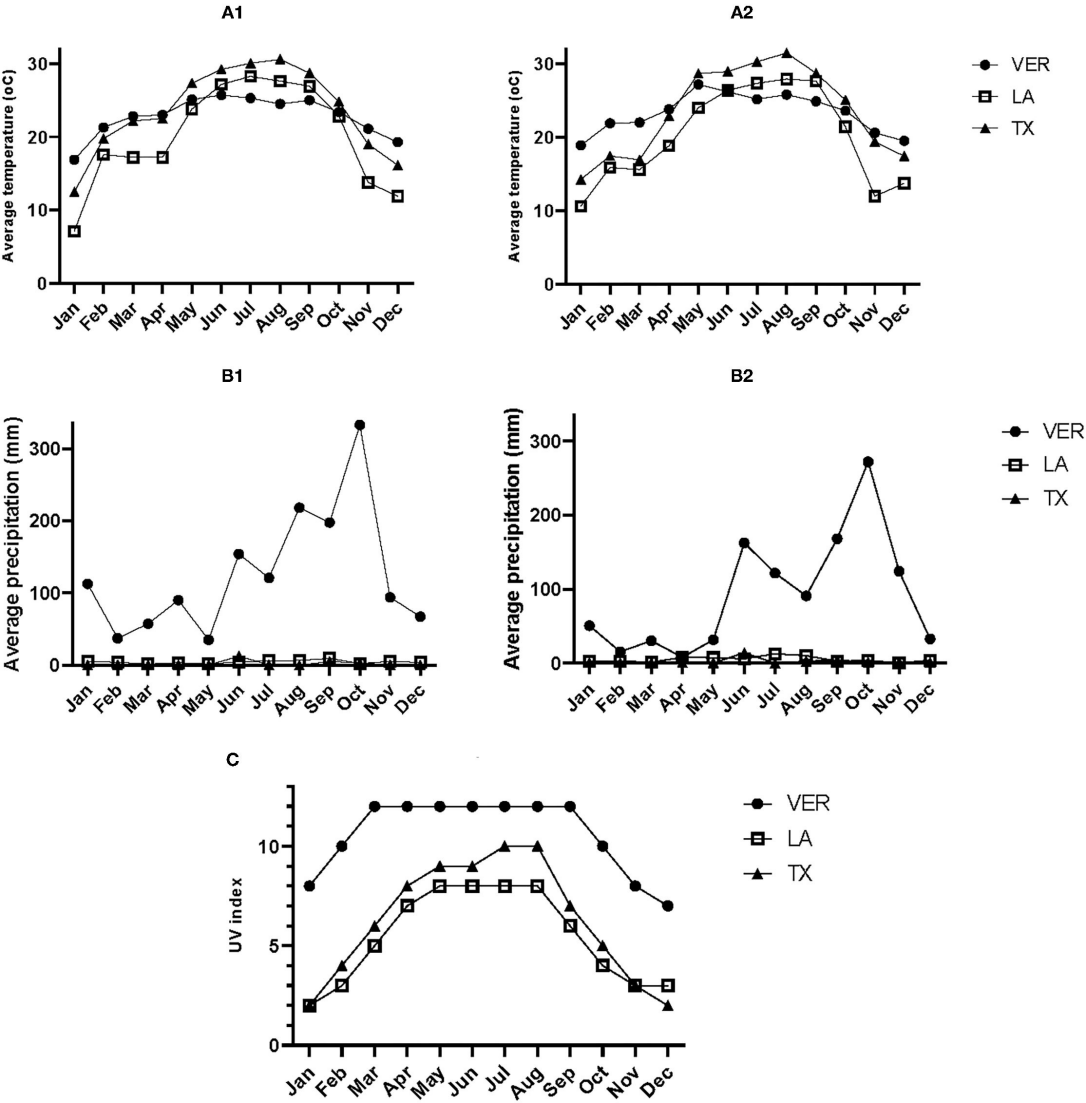
**(A1)** Temperature (oC) in 2018, **(A2)** temperature (oC) in 2019, **(B1)** precipitation (mm) in 2018, **(B2)** precipitation (mm) in 2019, **(C)** UV index in 2019. Records registered in three different locations of sugarcane production. VER: Mahuixtlan, Ver; MX, LA: St. Mary, LA, US.; TX: Santa Rosa, TX, US.

The lignin content (Table 1) in all three samples was significantly different; the VER sample contained more lignin than the other two samples. However, the lignin content was unrelated to the temperature during each sample's growth and harvest season. Based on the annual average temperatures when the samples were retrieved (Figure 3), none of the samples appeared under cold stress. The LA sample was exposed to temperatures below 10°C during January 2018 and 2019, probably increasing the ethanol soluble carbohydrates (ESC) or simple sugar values (Table 1) as a cryoprotectant measure. The optimum temperature for sugarcane growth and to retrieve stems for obtaining raw sugar is at a daily average of 22–30°C. However, the desired temperature for enhancing the sucrose content is 10–20°C (27). Low temperatures can induce cold stress on secondary metabolites. Therefore, the plant synthesizes cryoprotectant compounds such as sugars and nitrogen-based compounds (28). Further, cold stress can also increase the production of PhCs and lignin to protect against freeze damage (28).

On the contrary, higher temperatures can reduce photosynthesis, and for some plants, this can increase the production of anthocyanins (34). However, the registered temperatures (Figure 3) during 2018 and 2019 were not high enough to affect the production of the selected secondary metabolites.

#### Drought Stress

The rainfall or precipitation levels during 2018 and 2019 (Figure 3) differed depending on the location. On average, VER had weekly precipitation of 29.16 mm in 2018 and 29.3 mm in 2019, whereas LA had 1.04 and 1.19 mm in the same year. The TX location had only 0.46 and 0.53 mm of average rainfall every week. The rainfall levels in TX could explain why the TX samples show an intermediate abundance of secondary metabolites (Figure 2). Based on the precipitation levels and production of SCB from each location, it is evident that the sugarcane was not under drought stress because of the irrigation practices used in each location to overcome the absence of rainfall. Therefore, an additional in-depth study is needed to study the effect of pH, salinity, and metal concentration in the water source used for irrigation on the factors studied.

Sugarcane requires 1,500–2,500 mm of water during the growing season or 25–50 mm each week (35). Drought stress is induced by a prolonged period with low rainfall or water deficit and can reduce the chlorophyll contents in the plant. Water deficit can also increase the content of flavonoids and phenolic acids in Salix tree leaves (36). The production of flavonoids helps protect the plant during this stress. However, this phenomenon can reduce the saponin content in plants.

#### Heavy Metal Stress and Nutrients

The sugarcane needs high levels of nitrogen and potassium and low phosphorus levels. Based on the values from Table 1, TX samples showed higher potassium concentrations than the LA and VER samples. However, the potassium levels were generally normal for sugarcane growth. Further, all three samples showed low phosphorus levels, consistent with the requirements of sugarcane. Accumulation of copper can help increase the production of secondary metabolites; for example, it has been reported to increase digitalin production (37). However, the levels of Copper (Table 1) among the present samples were significantly different. The VER sample had a higher concentration with 54.7 ppm than the low values of 3.0 ppm in the LA sample and 5.33 ppm in the TX sample, which probably contributed to the increased abundance of some secondary metabolites.

The manganese content can also increase secondary metabolite production and antioxidant activity (38). In one study, different concentrations of this mineral were supplied to samples of *Mentha aquatica*. This addition to the concentration of manganese increased the secondary metabolite abundance and the total superoxide dismutase (SOD), catalase (CAT), and peroxidase (POX) activities (38). The manganese values were significantly different among the three samples in this study. The VER sample showed a higher concentration with 93.3 ppm, followed by LA with 79.3 ppm and TX with 52.33 ppm, explaining the differences in the abundance of secondary metabolites (Figure 2).

#### Ultraviolet Radiation

Sugarcane requires a fair amount of solar light. According to Figure 3, the UV index reached values of 11 during most of 2019 in VER, whereas TX reached a UV index of 11 during July and August. LA had a UV index of 8 between May and August. In general, all three locations had sufficient solar radiation to grow sugarcane, but the differences in the abundance of secondary metabolites could be related to the UV index observed during 2019.

According to NASA, UV light presents wavelengths shorter than visible light. This radiation from the sun is divided into UV-A, UV-B, and UV-C, where the most harmful is UV-C, which is entirely absorbed by the atmosphere (39). Most of the plants are UV resistant and use this radiation to their benefit by stimulating secondary metabolite production. For example, the effect of UV radiation on anthocyanin production was studied in sweet cherries and was found to increase flavonoids while decreasing the chlorophyll content (40, 41).

### Antimicrobial Activity of Sugarcane Bagasse Extracts

The antibiotic KAN inhibited the growth of each of the five bacterial strains to near 100%, even at low concentrations of 25 μg/mL. The antibiotic AMP was very effective against *E. coli K12* and *S. aureus* at the same concentration (Table 2). Further, the effect of AMP against *E. cloacae* and *E. aerogenes* was significant at 400 μg/mL. However, it showed only 10% inhibition of *S. aureus* at the highest concentration used in this study.

**Table 2:** Minimum inhibitory concentration of antibiotics and SCB extracts against bacteria strains (μg/mL).

**Sample**	**Bacterial strains**				
	***E. coli K12***	***E. cloacae***	***E. aerogenes***	***B. cereus***	***S. aureus***
KAN	25	25	25	25	25
AMP	25	400	400	25	–
VER	–	–	–	200	800
LA	–	–	–	–	–
TX	–	–	–	800	800

*SCB, Sugarcane bagasse; KAN, Kanamycin; AMP, Ampicillin; VER, Veracruz; LA, Louisiana; TX, Texas; –, No inhibition*.

The SCB extracts showed different effects depending on the geographical origin of the sample (Table 2). The most effective sample was VER, which showed 90% inhibition against *B. cereus* at 200 μg/mL and 50% inhibition against *S. aureus* at 800 μg/mL. The TX sample showed 50% inhibition against the same bacterial strains at 800 μg/mL. In comparison, the LA samples did not show more than 10% inhibition of any bacterial strain at the highest concentration of 800 μg/mL.

Against modified yeast, AMP showed only 20% growth inhibition at 800 μg/mL (Table 3). KAN showed 50% growth inhibition against SGS1 at 800 μg/mL but showed only 20% growth inhibition at the same concentration against the other yeast strains.

**Table 3:** Minimum inhibitory concentration of antibiotics and SCB extracts against modified yeast strains (μg/mL).

**Sample**	**Modified yeast strains**			
	***SGS1***	***RAD14***	***MLH1***	***MGT1***
KAN	800	–	–	–
AMP	–	–	–	–
VER	800	800	800	–
LA	800	800	800	–
TX	800	800	800	–

*SCB, Sugarcane bagasse; KAN, Kanamycin; AMP, ampicillin; VER, Veracruz; LA, Louisiana; TX, Texas; –, No inhibition*.

The SCB extracts generally affected the growth of SGS1, RAD14, and MLH1 yeast strains at 800 μg/mL concentrations. The VER and TX samples were the most effective against SGS1, with more than 80% growth inhibition, whereas the LA sample showed 60% growth inhibition. Against RAD14, the VER sample was the most effective with 50% growth inhibition (Table 3). The SCB extracts showed a similar MIC (200 μg/mL) against *B. cereus* and lower (800 μg/mL) against *S. aureus* compared with the phenolic extracts from annatto seeds and leaves (19). The MIC of 800 μg/mL was comparable with the MIC shown by the crude methanolic extract from *Peperomia galioides* against *S. aureus* (42).

Overall, the SCB extracts showed important antimicrobial properties that can be used as a potential nutraceutical or food additive. The effects against bacterial strains *B. cereus* (MIC = 200 μg/mL) and *S. aureus* (MIC = 800 μg/mL) can be beneficial because these bacteria are related to food poisoning. In addition, the effects against SGS1 RAD14, and MLH1 yeast strains (MIC = 800 μg/mL), offer an opportunity for future research using actual carcinogenic cells because these yeast strains harbor mutations relevant to the Bloom and Werner syndrome (SGS1), XPA human homolog (RAD14), and colon, ovary, or renal cancer (MLH1) (43–45).

## Conclusions

The samples' phenolic content and antioxidant activity remained stable across different extraction processes from the three locations. However, the SCB from the three locations showed differences in the abundance of secondary metabolites. Such differences are related to the UV radiation, nutrient components, temperature, and water stress present in the different geographical regions. The bioactive compounds in SCB have been previously reported as therapeutic and anticarcinogenic agents. SCB extracts showed promising effects against well-known pathogenic bacteria. In addition, they may possess possible anticancer activity based on their inhibitory effects on yeast strains harboring mutations relevant to various cancer types. Overall, this study provides a baseline to understand the potential benefits of using SCB as a source of phenolic compounds. To our knowledge, few studies have identified these compounds in SCB and indicated their antimicrobial activities along with their microencapsulation processing. These findings indicate the potential of using SCB as a potential source of food additives or nutraceuticals, in addition to its value as an industrial byproduct with easy availability.

Further studies are required to develop a food additive or nutraceutical from SCB phenolic compounds. Also, test the anticarcinogenic properties against real carcinogenic cell lines and conduct *in vivo* tests. The microencapsulation analysis of the extracted bioactive compounds is in preparation along with this study. Further investigation is necessary to determine the bioavailability of microencapsulated bioactive compounds by *in vitro* digestibility assays and their final nutrient composition.

## Data Availability Statement

The original contributions presented in the study are included in the article/Supplementary Material, further inquiries can be directed to the corresponding author/s.

## Author Contributions

VV-M designed and interpreted the results and collected test data. ED did study design, writing, statistical analysis, and interpretation of the results. DV-R worked on statistical analysis. LR-U, JL-C, and HL-C designed and interpreted the results. All authors contributed to the article and approved the submitted version.

## Funding

This work was supported in part by the USDA National Institute of Food and Agriculture, (Hatch grant (1010849) Food Bioengineering Technology of Agro-industrial Products and ALFA-LoT-Alliance for Smart Agriculture in the Internet of Things Era Award Number 2018-38422-28564), Cotton Incorporated grant (13-855) Utilization of Food Processing Technology to Add Value to Cotton as a Food Crop, the USDA-AFRI-30781, and Agriculture and Food Research Initiative-Agricultural Workforce Training Priority Area (grant no. 2021-67037-10692/project accession no. 1025548) from the U.S. Department of Agriculture, National Institute of Food and Agriculture.

## Conflict of Interest

The authors declare that the research was conducted in the absence of any commercial or financial relationships that could be construed as a potential conflict of interest.

## Publisher's Note

All claims expressed in this article are solely those of the authors and do not necessarily represent those of their affiliated organizations, or those of the publisher, the editors and the reviewers. Any product that may be evaluated in this article, or claim that may be made by its manufacturer, is not guaranteed or endorsed by the publisher.
