# Supplementary material for: Effect of Different Extraction Methods and Geographical Origins on the Total Phenolic Yield, Composition, and Antimicrobial Activity of Sugarcane Bagasse Extracts
Source: Front Nutr. 2022 Feb 24;9:834557. doi: 10.3389/fnut.2022.834557 (PMC8908448; doi:10.3389/fnut.2022.834557)
Supplement: Supplementary file 1 [file Table_1.DOCX]

**Table .** Central composite experimental design of phenolic compounds extraction from sugarcane bagasse.

| Block | Factor 1  Solvent | Factor 2  Extraction method | Response 1  TPC mg GAE/gr |
| --- | --- | --- | --- |
| 1 | A | X | 2.91 |
| 1 | B | Z | 6.10 |
| 1 | A | X | 2.71 |
| 1 | A | Z | 5.84 |
| 1 | A | X | 2.81 |
| 1 | A | X | 3.02 |
| 1 | A | Y | 5.43 |
| 1 | A | Y | 5.43 |
| 1 | A | Z | 6.25 |
| 1 | A | X | 2.96 |
| 1 | B | X | 3.07 |
| 1 | B | Y | 6.00 |
| 1 | A | Y | 5.69 |
| 1 | A | X | 2.71 |
| 1 | A | Y | 5.64 |
| 1 | B | Z | 5.95 |
| 1 | A | X | 2.81 |
| 1 | B | Y | 5.89 |
| 1 | B | X | 3.02 |
| 1 | A | Z | 5.89 |
| 1 | B | Z | 5.89 |
| 1 | A | Z | 6.20 |
| 1 | B | X | 3.12 |
| 1 | B | Y | 6.00 |
| 1 | A | X | 2.96 |
| 1 | A | Y | 5.89 |
| 1 | A | Z | 6.00 |
| 1 | A | Y | 5.74 |
| 1 | B | Z | 5.84 |
| 1 | B | Z | 5.74 |
| 1 | A | Z | 6.05 |
| 1 | B | Z | 5.89 |
| 1 | A | Y | 5.79 |
| 1 | A | Z | 6.00 |
| 1 | A | X | 2.96 |
| 1 | B | X | 3.12 |
| 1 | B | Y | 5.84 |
| 1 | B | X | 3.43 |
| 1 | B | Z | 5.89 |
| 1 | A | X | 2.71 |
| 1 | B | Y | 6.10 |
| 1 | A | Y | 5.48 |
| 1 | A | Y | 5.79 |
| 1 | B | Y | 6.20 |
| 1 | A | Y | 5.74 |
| 1 | B | X | 3.17 |
| 1 | B | X | 3.17 |
| 1 | B | Z | 6.36 |
| 1 | A | X | 2.91 |
| 1 | B | Z | 5.89 |
| 1 | A | X | 3.02 |
| 1 | A | Z | 5.84 |
| 1 | A | Y | 5.79 |
| 1 | B | Y | 6.20 |
| 1 | B | X | 3.32 |
| 1 | B | Y | 5.89 |
| 1 | B | Y | 5.89 |
| 1 | B | Y | 6.10 |
| 1 | A | Z | 5.84 |
| 1 | A | Z | 6.15 |
| 1 | A | Y | 5.95 |
| 1 | B | X | 3.27 |
| 1 | B | Y | 5.89 |
| 1 | B | Z | 6.25 |
| 1 | A | Z | 6.15 |
| 1 | B | X | 3.32 |
| 1 | A | Z | 6.05 |
| 1 | B | X | 3.27 |
| 1 | B | Z | 6.10 |
| 1 | B | X | 3.32 |
| 1 | B | Z | 6.10 |
| 1 | B | Y | 6.00 |
| 2 | A | Z | 6.82 |
| 2 | B | Z | 7.23 |
| 2 | B | Z | 7.54 |
| 2 | A | Z | 6.72 |
| 2 | B | X | 4.66 |
| 2 | A | Z | 7.08 |
| 2 | A | X | 4.10 |
| 2 | B | X | 4.66 |
| 2 | B | Y | 6.82 |
| 2 | B | Z | 7.28 |
| 2 | A | Y | 6.56 |
| 2 | A | X | 3.94 |
| 2 | A | Y | 6.67 |
| 2 | B | Y | 6.77 |
| 2 | B | X | 4.51 |
| 2 | A | Z | 6.87 |
| 2 | A | X | 4.10 |
| 2 | B | Z | 7.28 |
| 2 | A | Y | 6.87 |
| 2 | A | Y | 7.03 |
| 2 | A | X | 4.10 |
| 2 | A | Y | 6.56 |
| 2 | B | X | 4.61 |
| 2 | B | X | 4.61 |
| 2 | B | X | 4.40 |
| 2 | A | Y | 6.41 |
| 2 | A | Y | 6.36 |
| 2 | A | X | 4.15 |
| 2 | A | Z | 7.13 |
| 2 | B | Y | 6.77 |
| 2 | A | Y | 6.41 |
| 2 | B | Y | 6.31 |
| 2 | A | X | 3.89 |
| 2 | B | Z | 6.97 |
| 2 | B | X | 4.35 |
| 2 | B | Y | 6.46 |
| 2 | B | Y | 6.31 |
| 2 | B | X | 4.45 |
| 2 | B | X | 4.51 |
| 2 | A | X | 4.15 |
| 2 | A | X | 4.10 |
| 2 | A | X | 4.15 |
| 2 | A | Z | 6.87 |
| 2 | B | X | 4.30 |
| 2 | A | X | 4.15 |
| 2 | A | X | 3.84 |
| 2 | A | Z | 6.87 |
| 2 | A | Z | 6.77 |
| 2 | A | Y | 6.97 |
| 2 | A | Z | 6.87 |
| 2 | B | Z | 7.03 |
| 2 | B | Y | 6.31 |
| 2 | B | Z | 7.03 |
| 2 | B | Y | 6.56 |
| 2 | B | Z | 7.13 |
| 2 | B | X | 4.20 |
| 2 | B | Y | 7.08 |
| 2 | A | X | 3.89 |
| 2 | B | Y | 7.03 |
| 2 | A | Y | 6.20 |
| 2 | A | Z | 6.87 |
| 2 | A | Z | 7.08 |
| 2 | A | Z | 7.13 |
| 2 | B | Y | 6.97 |
| 2 | A | Y | 6.25 |
| 2 | B | X | 4.61 |
| 2 | B | Z | 7.03 |
| 2 | B | Z | 7.08 |
| 2 | A | Y | 6.61 |
| 2 | B | Z | 7.03 |
| 2 | B | Y | 7.13 |
| 2 | B | Z | 7.54 |
| 3 | B | X | 3.48 |
| 3 | B | X | 3.53 |
| 3 | B | Y | 5.84 |
| 3 | A | Z | 6.00 |
| 3 | A | X | 3.17 |
| 3 | B | Y | 5.89 |
| 3 | B | Y | 5.84 |
| 3 | A | X | 3.22 |
| 3 | A | Y | 5.89 |
| 3 | A | Y | 5.79 |
| 3 | A | Z | 6.05 |
| 3 | A | X | 3.22 |
| 3 | B | Y | 5.95 |
| 3 | A | Y | 5.69 |
| 3 | B | Z | 6.20 |
| 3 | A | X | 3.12 |
| 3 | A | Z | 6.05 |
| 3 | B | Y | 6.31 |
| 3 | A | Z | 5.89 |
| 3 | A | Y | 5.69 |
| 3 | B | X | 3.43 |
| 3 | A | Y | 5.53 |
| 3 | B | X | 3.58 |
| 3 | B | Z | 6.10 |
| 3 | A | X | 3.22 |
| 3 | A | X | 3.43 |
| 3 | A | Z | 5.74 |
| 3 | B | Z | 6.20 |
| 3 | B | Y | 5.95 |
| 3 | B | Z | 6.15 |
| 3 | B | Y | 5.95 |
| 3 | A | Y | 5.48 |
| 3 | B | X | 3.53 |
| 3 | A | Y | 5.48 |
| 3 | A | X | 3.22 |
| 3 | B | Z | 6.10 |
| 3 | A | Z | 5.95 |
| 3 | B | Y | 6.46 |
| 3 | A | Y | 5.84 |
| 3 | A | X | 3.17 |
| 3 | B | Z | 6.00 |
| 3 | B | Z | 5.89 |
| 3 | B | Z | 6.20 |
| 3 | A | Z | 5.89 |
| 3 | B | X | 3.68 |
| 3 | B | Z | 6.31 |
| 3 | B | Y | 6.31 |
| 3 | A | Z | 5.74 |
| 3 | B | Z | 6.25 |
| 3 | B | X | 3.43 |
| 3 | B | X | 3.43 |
| 3 | A | Y | 5.69 |
| 3 | B | X | 3.68 |
| 3 | A | Y | 5.74 |
| 3 | B | Y | 5.84 |
| 3 | A | X | 3.12 |
| 3 | B | X | 3.43 |
| 3 | B | Z | 6.36 |
| 3 | B | X | 3.48 |
| 3 | B | Y | 5.74 |
| 3 | B | X | 3.48 |
| 3 | A | X | 3.32 |
| 3 | A | X | 3.22 |
| 3 | A | Z | 5.74 |
| 3 | A | Y | 5.79 |
| 3 | A | Y | 5.84 |
| 3 | A | X | 3.07 |
| 3 | B | Z | 6.31 |
| 3 | A | Z | 5.84 |
| 3 | B | Y | 6.20 |
| 3 | A | Z | 5.79 |
| 3 | A | Z | 5.84 |

Block: 1= VER, 2= LA, 3= TX. Solvent; A= 90% ethanol, B= 90% methanol. Extraction method: X= Sonication 30 min, Y= Incubation/shaker 24 h, Z= Incubation/shaker 48 h
